# Pastoralism and delay in diagnosis of TB in Ethiopia

**DOI:** 10.1186/1471-2458-9-5

**Published:** 2009-01-07

**Authors:** Abdi A Gele, Gunnar Bjune, Fekadu Abebe

**Affiliations:** 1 Institute of General Practice and Community Medicine, Faculty of Medicine, University of Oslo, Oslo, Norway

## Abstract

**Background:**

Tuberculosis (TB) is a major public health problem in the Horn of Africa with Ethiopia being the most affected where TB cases increase at the rate of 2.6% each year. One of the main contributing factors for this rise is increasing transmission due to large number of untreated patients, serving as reservoirs of the infection within the communities. Reduction of the time between onset of TB symptoms to diagnosis is therefore a prerequisite to bring the TB epidemic under control. The aim of this study was to measure duration of delay among pastoralist TB patients at TB management units in Somali Regional State (SRS) of Ethiopia.

**Methods:**

A cross sectional study of 226 TB patients with pastoralist identity was conducted in SRS of Ethiopia from June to September 2007. Patients were interviewed using questionnaire based interview. Time between onset of TB symptoms and first visit to a professional health care provider (patient delay), and the time between first visits to the professional health care provider to the date of diagnosis (medical provider's delay) were analyzed. Both pulmonary and extrapulmonary TB patients were included in the study.

**Result:**

A total of 226 pastoralist TB patients were included in this study; 93 (41.2%) were nomadic pastoralists and 133 (58.8%) were agro-pastoralists. Median patient delay was found to be 60 days with range of 10–1800 days (83 days for nomadic pastoralists and 57 days for agro-pastoralists). Median health care provider's delay was 6 days and median total delay was 70 days in this study. Patient delay constituted 86% of the total delay. In multivariate logistic regression analysis, nomadic pastoralism (aOR. 2.69, CI 1.47–4.91) and having low biomedical knowledge on TB (aOR. 2.02, CI 1.02–3.98) were significantly associated with prolonged patient delay. However, the only observed risk factor for very long patient delay >120 days was distance to health facility (aOR.4.23, CI 1.32–13.54). Extra-pulmonary TB was the only observed predictor for health care providers' delay (aOR. 3.39, CI 1.68–6.83).

**Conclusion:**

Patient delay observed among pastoralist TB patients in SRS is one of the highest reported so far from developing countries, exceeding two years in some patients. This long patient delay appears to be associated with patient's inadequate knowledge of the disease and distance to health care facility with nomadic pastoralists being the most affected. Regional TB control programmes need to consider the exceptional circumstances of pastoralists, to maximise their access to TB services.

## Background

Tuberculosis is one of the major causes of morbidity and mortality in the Horn of Africa with Ethiopia carrying a heavy burden [1]. The most important component of TB control is prompt detection and treatment of patients with active TB [2]. Prolonged delay of such patients to treatment may lead to more advanced disease, high mortality, and enhance continual transmission in the community. Diagnostic delay of longer than two months have been indicated to spread the disease to domestic contacts [3]. Reduction of the time between onset of TB symptoms to diagnosis is therefore a prerequisite to bring TB epidemic under control [4]. Diagnostic delay reflects patient delays in seeking health care, health care providers delay in making prompt and correct diagnosis and initiation of treatment [5]. Identifying where delays occur and the reasons for the delay help TB control programs improve their control strategies [6].

Ethiopia is the third most populous country in Africa and number 8 of the countries with highest TB burden in the world. Long delay of TB patients in diagnosis pose a formidable challenge to TB control in Ethiopia [7-10]. This has been attributed to a number of factors, mainly long distance to health facilities [7-9], limited awareness of TB disease [7-9] and prevalent use of traditional healers [7,8].

Pastoralists are migratory people whose livelihood largely depends on livestock raising. An estimated 50–100 million that live in the developing world, 60% are in sub-Saharan Africa. In the Horn of Africa, pastoralists constitute 70% of general population in Somalia, 33% in Eritrea, 20% in Kenya and Djibouti, 12% in Ethiopia and 60% of rural populations in Sudan [11]. In these countries, pastoralists often dwell in border areas; highly volatile and insecure environments that is often beyond the reach of health services. Accordingly, disease control activities including TB control programmes are often absent in those areas [12] or are not well adapted to pastoralists' mobile lifestyle [13]. Although a large number of studies about diagnostic delay of TB have been conducted in the Horn of Africa, particularly in Ethiopia, researchers and health planners have so far neglected pastoralist communities. Thus, the extent of delay in diagnosis of pastoralist TB patients in the region as well as factors underlying their delay remains largely unknown. The aim of this study was to determine patterns of delay among pastoralist TB patients at TB management units in SRS of Ethiopia.

### Study area

The SRS is the second largest among the nine regions of Ethiopia, with land area of 375, 000 km^2 ^and estimated population of 4 million. Three different livelihood systems exist in the SRS namely; pastoralism, agro-pastoralism and urban [14]. An estimated 85% of regional populations practice the former two livelihood systems.

The people in the SRS are among the poorest in Ethiopia, and thus they are disproportionately affected by TB. The annual incidence of smear positive pulmonary TB in the year 2000 was noted to be 175–250/100000, which is higher than the national level of 165/100000 [15]. Regional TB control program adopted the DOTS strategy which is implemented through DOTS clinics that are located in major towns. Private sector is very rare in SRS. However, neither the private sector nor traditional healers are involved in regional TB control program.

## Methods

An institution based, cross sectional study was carried out in SRS of Ethiopia. The study population was selected from the TB management units in Jigjiga and Shinile zones of the region. The selection of the study site was based on accessibility in terms of security and infrastructure such as roads. Moreover, about 50% of diagnostic facilities in the region are found within these two zones, therefore pastoralists from other zones in the region come for diagnosis and treatment there. Because of being illiterate, witnessed consent was obtained from patients. The study was ethically cleared by Norwegian Ethical Committee and Ethiopian Science and Technology Commission.

Pre-tested structured questionnaires were consecutively administered to 226 patients with pastoralist identity who had been in the intensive phase of TB treatment from June to September 2007. Patients over 15 yrs of age, who gave informed consent, were included in the study, while treatment failures, relapse case and non pastoralists were excluded.

The questionnaires included socio-demographic details as well as questions that assess patients' knowledge on TB. Questions addressed the cause of TB, curability of TB, if patients were aware that TB treatment is available free of charge and their awareness of transmission mechanisms of TB with emphasis on bovine TB. Patients were asked about major symptoms of TB, duration of major presenting symptoms, date of first visit to professional health care provider and date of diagnosis. Along with the questionnaire based interviews, we simultaneously crosschecked subjects' out-patient cards, patients' registration cards, laboratory registrations and TB registration books.

### Data analysis

SPSS (Statistical Package for the Social Sciences) version 14 was used for data analysis. This study investigated knowledge of patients on TB. In this case we posed 12 questions on causes, treatment and transmission mechanisms of TB. Afterwards, scoring system was designed and answering 5 out of the 12 questions correctly was taken as cut-off point between good and poor biomedical knowledge on TB [16].

Different cut-off points were used in evaluating duration of delay. In some studies a panel of experts agreed on 30 days as an acceptable delay [17,18] while majority of the studies used median of the observed data as a cut off point [7,8]. The present study adopted the later.

As data was not normally distributed, nonparametric tests such as Mann-Whitney and Kruskal-Wallis tests were employed in calculating group differences. In order to adjust confounding effect of several identified determinants of diagnostic delay, and to finally establish factors that may independently be associated with the delay, multivariate logistic regression analysis was performed. The association of predictor variables with the delays was assessed by using 95% confidence interval (CI) and adjusted odd ratio (aOR). *P*-value < 0.05 was considered statistically significant.

### Operational definitions

#### Patient delay

The time between the onset of clinical symptoms of TB to first visit to professional health care provider.

#### Medical providers' delay

The time from patients' first consultation with the professional health care provider for the symptoms of TB until the date of diagnosis.

#### Total delay

Sum of the patient delay and the medical providers' delay.

#### Nomadic pastoralists

People whose source of livelihood is livestock with which they move seasonally in search of pasture and water.

#### Agropastoralists

People whose main source of livelihood is livestock, but also practice small scale farming for additional income.

#### Health care provider

A professional medical practitioner licensed to treat illness and acting within the scope of that license. This includes doctors, health officers and nurses, while traditional health providers such as traditional healers and religious leaders are excluded.

#### Distance to health facility

Distance in km from patient's residence, at the time when patient took the decision of seeking medical care, to health care facility (excluding traditional health facility) where patients sought help first for current illness.

## Results

Two hundreds and twenty six TB patients were interviewed during June to September 2007. Number of males in this study was higher than that of females with ratio of 1.21:1. The mean age of study population was 32.2 ± 13.0 SD. High proportions of study participants (85%) were in the age group between 16–45 yrs old. Majority of study population (200, 88.5%) were illiterates. The number of agro-pastoralists (133, 58.8%) exceeded that of nomadic pastoralists (93, 41.2%). The median distance of subject's residence to health facility, at the time of decision to seeking care, was 24 km, ranging 16 km for agro-pastoralists to 36 km for nomadic pastoralists. Higher proportion of the participants had low biomedical knowledge on TB (145, 64.2%). Majority of respondents (87%) sought traditional health care first for current illness. Regarding form of TB, 175 (77.4%) patients had pulmonary TB and 51 (22.6%) had extra-pulmonary TB (EPTB). Among pulmonary TB patients, 124 (71%) were smear positive and 51 (29%) were smear negative. The majority of smear positive pulmonary TB patients (74.5%) had high sputum grading (3+) while only 3.2% had scant (1+) and 28 (22.3%) had moderate (2+).

### Patient delay

The median patient delay of 60 days and mean delay of 130 days was found (10^th ^and 90^th ^percentiles were 30 and 341 days, respectively). The longest patient delay observed was 1800 days.

Median patient delay of 83 days was observed among nomadic pastoralists which was significantly higher than the median patient delay of 57 days observed among agro-pastoralists (Mann-Whitney test, P < 0.001). Delay in diagnosis in relation to patient's pastoralist status is shown in table 1. Five patients with the longest delay exceeding two years were all nomadic pastoralists. Significant difference was also observed among those categorized as having high biomedical knowledge on TB and those with low biomedical knowledge on TB, with median patient delay difference of 13 days (Mann-Whitney test, P < 0.002). There was no significant difference with regard to patient delay by sex, education, age, first health seeking action and form of TB.

**Table 1:** Patient delay in diagnosis of TB in relation to patients' pastoralist status

Delay	Nomadic pastoralists		Agro-pastoralists		Total	
Days	N	%	N	%	N	%

0–60	39	41.9	88	66.2	127	56.2

61–120	32	34.4	23	17.3	55	24.3

121–360	14	15	17	12.8	31	13.7

>360	8	8.6	5	3.7	13	5.7

**Total**	93	100	133	100	226	100

Multivariate logistic regression analysis showed that low biomedical knowledge of TB (aOR. 2.02, CI 1.02–3.98) and nomadic pastoralism (aOR.2.69, CI 1.47–4.91) were independent predictors for long patient delay (table 2).

**Table 2:** The association of socio-cultural factors with patient delay.

Variables	Patient delay >60 days	Patient delay ≤60 days	Crude OR (95% CI)	Adjusted OR (95% CI)
**Gender**				

Male	52	72	1.00	1.00

Female	47	55	0.698(0.69–2.06)	1.01 (0.55–1.86)

**Marital status**				

Married	68	61	1.00	1.00

Single	23	56	0.72(0.26–1.93)	0.68 (0.23–2.02)

Widowed or divorced	8	10	1.95(0.68–5.55)	1.57 (0.46–5.37)

**Education**				

Illiterate	92	108	1.00	1.00

Literate	7	19	2.31(0.93–5.74)	1.55 (0.47–5.05)

**Occupation**				

Pastoralist	94	108	1.00	1.00

With supplementary Job	5	19	0.30(0.10–084)	0.53 (0.15–1.82)

**Type of pastoralist**				

Agro-pastoralists	45	88	1.00	1.00

Nomadic pastoralists	54	39	2.71(1.57–4.68)	2.69 (1.47–4.91) *

**Knowledge on TB**				

Good biomedical knowledge on TB	24	57	1.00	1.00

Low biomedical knowledge on TB	75	70	2.54(1.43–4.53)	2.02 (1.02–3.98) *

**Distance to health facility**				

Distance ≤10 km	18	45	1.00	1.00

Distance >10 km	81	82	0.40(0.22–0.76)	0.63(0.30–1.30)

**First health seeking action**				

Traditional means	89	108	1.00	1.00

Modern health care	10	19	1.57(0.70–3.54)	1.12 (0.45–2.17)

*Significant at <0.05

Adjusted: Gender, Age, Marital status, education, occupation, first health seeking action, distance to health facility and form of TB.

Since 20% of study subjects had median patient delay of over 120 days, we investigated characteristics that might have been associated with such a long patient delay using 120 days as cut off point for comparison. In multivariate logistic regression analysis, distance to health facility proved to be a significant risk factor for patient delay over 120 days (aOR.4.23, CI 1.32–13.54).

### Health care providers' delay

In this study median provider's delay of 6 days with mean delay of 9 days was found (10^th ^and 90th percentiles were 2 and 19 days, respectively). The median provider's delay of 10.2 days was observed among extrapulmonary TB patients, which was significantly higher than the median provider's delay of 5.8 days observed among pulmonary TB patients (Mann-Whitney test, P < 0.002). However, 85% of the patients were diagnosed within fifteen days. Multivariate logistic regression analysis indicated that extrapulmonary TB was the only predictor for long health care providers' delay (aOR. 3.39, CI 1.68–6.83).

### Total delay

Median total delay of 70 days with mean of 140 days was found (10^th ^and 90^th ^percentiles were 34 and 345 days respectively). The highest delay observed was 1803 days. However, 17.7% of study respondents had total delay that exceeded 6 months, while almost 10% of study population had total delay that exceeded one year.

## Discussion

The present study is the first of its kind to report diagnostic delay of TB among pastoral communities in Africa, and it reveals an extremely long diagnostic delay of TB in pastoralist contexts.

We found a median patient delay of 60 days with a mean of 130 days, ranging from 10 days to 1800 days. This is one of the longest patients delay being reported so far from developing countries. Around 20% of the patients had delay that exceeds 120 days and 50% of them had pulmonary TB with high sputum grade. This is a serious concern due to the fact that each of these patients dispenses up to 3,500 bacilli in each cough, and may infect 10–15 people each year, eventually creating a public health time bomb.

The reported median patient delay was much higher than that reported from Amhara region of Ethiopia (median, 30 days) [7], Awasa, Ethiopia (median, 4.3 weeks) [8], Vietnam (median, 4 weeks) [5], and the seven countries of the Eastern Mediterranean Region including Iran (median, 24 days), Iraq (median, 31 days), Pakistan (median, 9 days), Syria (median, 31 days), Yemen (median, 28 days), Egypt (median, 12 days) and Somalia (median, 53 days) [19].

These studies targeted settled populations and therefore the longer patient delay of the present study could be explained by the distinctive socio-culture and lifestyle of pastoralists, often characterized with mobility, poverty and illiteracy compounded with lack of access to modern medical care. Earlier studies found that rural residence was a risk factor for diagnostic delay just because of poor access to health care and low awareness of TB disease among those populations [20,21]. This study documents an extremely long distance to health facilities (mean, 79.2 km) for nomadic pastoralists. This makes it clear that pastoralists are even more marginalized than rural sedentary populations regarding access to health care.

Low biomedical knowledge about the disease proved to be a significant predictor for patient delay. This result is in consistence with previous studies conducted in Ethiopia [7,9] and Tanzania [22]. Poor knowledge and practices related to TB disease was also reported from pastoralist communities in Tanzania [23]. Limited knowledge on TB was reported to encourage people to consider various traditional alternatives [24,25]. However, good lay knowledge on TB is prerequisite for early seeking of medical care.

Nomadic pastoralism was also found to be an independent predictor of patient delay. In this regard, nomadic pastoralists had significantly higher median patient delay (83 days) than agro-pastoralists (57 days). It is probably because nomadic pastoralists dwell in very remote areas, often far from health facilities particularly DOTS services. An earlier study in Ethiopia found significant association between distance to health facility and patient delay [9]. Access to health care is defined in Ethiopia as living within 10 km radius to health facility [26]. From that perspective, almost 84% of nomadic pastoralists in this study lacked access to health care, and therefore may rely on traditional health care with consequent patient delay. Somali pastoralists in Kenya were reported to prefer traditional health over modern medical care. The reason was because traditional healers were easily accessible for them whereas modern health facilities were either hardly accessible due to long distance or else, lacked the necessary services [27]. This could be true in SRS where 87% of the study participants sought traditional health care first for current illness.

The median distance to health facilities, where patients sought care first for the current illness, was 24 km. Earlier study in the same area has reported a similar figure [14]. However, this median distance was over two fold higher for nomadic pastoralists (36 km) than agro-pastoralists (16 km). Similar distance to health facilities was reported from pastoralists in northern Kenya [28]. Widespread poverty compounded with resource allocation bias in many African countries resulted in concentration of investment in urban areas, despite the fact that large proportions of their population are rural. Nonetheless, access to health care for all was a vital part of health related Millennium Development Goals adopted by the international community in 2000 [29]. Pastoralists in sub-Saharan Africa were long known to be vulnerable to exclusion from modern medical care [12,30]. We argue that this goal may not be achieved as long as large segments of the population are deprived access to health care just because they live under extreme conditions.

Regarding providers' delay, median of 6 days and mean of 9 days was found. This was lower than that reported from Ghana (median, 8 weeks) [31] but similar to the findings of studies in Addis Ababa, Ethiopia (median, 6 days) [9]. The low providers' delay in this study was probably due to high degree of alertness on the side of health workers to suspect TB, which is an event quite common in TB endemic areas [9]. Moreover, pastoralists were reported to seek professional health services only with advanced stage of illness [13]. Therefore, the low median provider's delay in this study could be due to presentation of patients to health facilities with advanced symptoms of the disease, which probably made its diagnosis easier.

Having EPTB was found to be the only predictor of long health care providers' delay. Earlier studies documented similar findings [6,32]. This study was conducted in an area where diagnosis of EPTB exclusively relies on clinical diagnosis. Nonetheless, extra pulmonary TB can involve almost any organ of the human body and therefore it might present with wide range of non-specific clinical manifestations. This might make it difficult for health care providers to rule out other diseases with comparable symptoms, hence failure to accurately diagnose the disease at the initial presentation.

Extrapulmonary TB constituted 22% of all cases in this study. Noteworthy, EPTB is more common in children [33] and patients with HIV infection [34]. However, children were excluded from this study and we lack data on HIV prevalence in SRS particularly among pastoral communities. However, as bovine TB more often leads to EPTB and is predominantly found in areas where people are in close contact with livestock [35], it is likely that bovine TB is highly prevalent among Somali pastoralists. This is supported by the fact that risk factors for bovine TB transmission such as consumption of raw milk and sharing same accommodation (same fence) with livestock were found highly prevalent among this community.

The present study has several limitations. The delays was assessed through self reporting, implying the possibility of a recall bias. Moreover, some of the patients had been on treatment for a month during interview, which further aggravates the recall bias. However, this is minimised by using seasonal and religious festivals to facilitate patients' recall, and moreover, qualitative probing questions were used for validation of the answers. The study was also limited in scope because it covered only two zones out of the nine zones of the region. It was a cross sectional study, thus limiting our ability to decide cause and effect relationship. The study was an institution based study and therefore we do not know the real burden of the delay in wider society. This study was conducted from June to September 2007. Because of the mobility nature of the study population, the season in which we conducted the study might have influenced the reported delay.

## Conclusion

Patient delay observed among pastoralist TB patients in SRS of Ethiopia is one of the highest in developing countries. This substantial patient delay to diagnosis is a major contributing factor for increasing transmission of TB in Ethiopia. Poor awareness of patients about the disease and pastoralist's limited access to health care, with nomadic pastoralists being the most affected, are the bottom-line in apparent diagnostic delay of pastoralist TB patients in SRS. Increased awareness of the disease is crucial in improving health seeking behaviour, but awareness alone may not be sufficient while diagnostic and treatment facilities are not within reasonable reach. Basic medical training should be considered to influential members in pastoralist communities, such as religious leaders and traditional healers, regarding detection of TB suspects, referral systems, drug distribution and observation and dissemination of health information such as causes of TB, its symptoms, how to treat it correctly and where to get this treatment. On the supply side, regular supervision and adequate drug supply should be granted by regional health authority. Participation of traditional healers in TB control has been widely advocated [36,37], and significant improvement was reported in many areas [38]. Involvement of traditional health providers in regional TB control programmes may therefore encourage early care seeking and adherence of TB patients to treatment with subsequent reduction of disease transmission in the region.

## Competing interests

The authors declare that they have no competing interests.

## Authors' contributions

AG designed the protocol, carried out the field work, did data analysis and drafted the manuscript. GB participated in the write up of the protocol, in the data analysis and write up of the manuscript. FA participated in the write up of the protocol, in the data analysis and write up of the manuscript. All authors read and approved the final manuscript.

## Pre-publication history

The pre-publication history for this paper can be accessed here:


